# Pure Endoscopic Orbital Decompression in Graves’ Orbitopathy: A Comprehensive Retrospective Analysis of Objective and Subjective Outcomes

**DOI:** 10.3390/medsci13040287

**Published:** 2025-11-27

**Authors:** Santiago Almanzo, Miguel Saro-Buendía, Inés Tortajada-Torralba, Cristina Peris-Moreno, Enrique España-Gregori, Miguel Armengot, Alfonso García-Piñero

**Affiliations:** 1 Department of Otorhinolaryngology, Hospital Universitari i Politècnic La Fe, 46026 Valencia, Spain; misabuen@alumni.uv.es (M.S.-B.); inestortor@gmail.com (I.T.-T.); cristinaperis15@gmail.com (C.P.-M.); miguel.armengot@uv.es (M.A.); garcia_alfpin@gva.es (A.G.-P.); 2 Department of Surgery, Faculty of Medicine and Dentistry, University of Valencia, 46010 Valencia, Spain; espana_enr@gva.es; 3 Department of Ophthalmology, Hospital Universitari i Politècnic La Fe, 46026 Valencia, Spain

**Keywords:** graves orbitopathy, orbital decompression, endoscopic surgery, proptosis, GO-QoL, quality of life

## Abstract

**Background and Objectives:** Graves’ orbitopathy (GO) is an autoimmune disease that can cause severe visual dysfunction and cosmetic impairment. Pure endoscopic orbital decompression reduces proptosis with minimal external morbidity. However, studies integrating both objective outcomes and patient-reported quality of life remain limited. This study aimed to analyze objective and subjective outcomes of pure endoscopic orbital decompression in inactive GO. **Materials and Methods:** We retrospectively reviewed 20 consecutive patients with severe inactive GO who underwent pure endoscopic transnasal orbital decompression between 2020 and 2023. Proptosis was measured using Hertel exophthalmometry, and quality of life was assessed with the disease-specific GO-QoL (Graves’ Ophthalmopathy Quality of Life) questionnaire (functional and appearance subscales). Minimum follow-up was 12 months. Pre- and postoperative changes were compared using paired *t* tests. **Results:** A total of 26 orbits were operated on. Mean proptosis decreased by 3.85 mm (*p* < 0.001). GO-QoL improved in the functional (+3.27, *p* < 0.001) and appearance (+5.77, *p* < 0.001) subscales. No complications or new/worsened diplopia were observed. **Conclusions:** Pure endoscopic orbital decompression is a safe and effective technique to reduce proptosis in inactive GO. Although quality-of-life scores improved significantly, the clinical relevance may vary, highlighting the need to integrate objective outcomes and patient perception when evaluating surgical results.

## 1. Introduction

Graves’ orbitopathy (GO), also known as thyroid eye disease, is the most common extrathyroidal manifestation of Graves–Basedow disease, occurring in approximately 25–50% of patients [1,2]. Its pathophysiology is based on an autoimmune reaction directed against orbital fibroblasts, which express the thyroid-stimulating hormone receptor and the insulin-like growth factor-1 receptor, triggering an inflammatory cascade with lymphocytic infiltration, tissue edema, increased muscle volume, and adipose tissue proliferation [1,2].

Clinically, GO presents with proptosis, eyelid retraction, diplopia, and, in severe cases, optic neuropathy. Its course is characterized by an active inflammatory phase and an inactive, fibrotic or “burnt-out” phase, the latter being the appropriate time for rehabilitative surgical interventions such as orbital decompression [2].

In the active phase, medical therapy includes intravenous corticosteroid pulses and orbital radiotherapy in selected cases. These modalities can improve ocular motility and diplopia and may stabilize disease [2,3,4]. Orbital decompression is therefore considered for vision-threatening disease, disfiguring proptosis, exposure keratopathy, or persistent dysfunction, and is preferably performed in the inactive phase [2].

Orbital decompression aims to increase the bony orbital volume to reduce proptosis and relieve compression of the optic nerve. Several surgical approaches exist: transantral, transpalpebral, coronal, and transnasal endoscopic. The latter has gained popularity due to its lower external morbidity and its ability to directly address the medial and inferior orbital walls [5,6]. The endoscopic technique, in addition to being safe and effective, has demonstrated both functional and esthetic benefits in patients with moderate-to-severe GO [3].

Historically, the outcomes of orbital decompression were assessed by the reduction in proptosis measured with exophthalmometry or imaging [7,8]. In recent years, however, increasing importance has been placed on patient-reported outcomes, particularly the perception of appearance and visual function. The GO-QoL (Graves’ Ophthalmopathy Quality of Life) questionnaire has been validated as a disease-specific tool to measure the impact of GO on quality of life [9,10] and is recommended by European guidelines [2].

Several studies have shown a significant improvement in quality of life after orbital decompression, even in the absence of marked objective changes, underlining the importance of integrating both types of evaluation [9,10,11]. Furthermore, evidence exists of a correlation between the degree of proptosis reduction and the improvement in esthetic perception [12].

In the international literature, series that combine pure endoscopic techniques with both objective (proptosis) and subjective (GO-QoL) evaluations remain scarce. To our knowledge, no studies have addressed both simultaneously, apart from partial analyses [6] such as that of Chu et al. This highlights the novelty and clinical relevance of our study within the current evidence landscape.

The aim of this retrospective, consecutive single-center study was to report outcomes of pure endoscopic transnasal orbital decompression in patients with severe inactive GO with prespecified 12-month follow-up, pairing objective (Hertel) and patient-reported (GO-QoL) measures and reporting effect sizes with 95% confidence intervals alongside significance testing to provide comparable magnitude estimates.

## 2. Materials and Methods

A retrospective observational study was conducted between January 2020 and December 2023 at the Department of Otorhinolaryngology of a tertiary university hospital. The study was conducted in accordance with the Declaration of Helsinki, and the protocol was reviewed and approved by the ethics committee of the principal investigators’ institution (Registry No.: 2025-0790-1) on 24 September 2025. The exemption from informed consent proposed for this study was approved by the ethics committee of our institution in accordance with local legislation (Law 14/2007, of 3 July, on Biomedical Research (Spanish Biomedical Research Act)).

Patients aged >18 years with a clinical and radiological diagnosis of inactive GO were included if they presented an indication for orbital decompression due to disfiguring proptosis, exposure keratopathy, incipient optic neuropathy, or functional symptoms associated with soft tissue retraction. Exclusion criteria were previous orbital surgery, non-thyroid-related orbitopathy, or inability to complete postoperative follow-up. Extraocular muscle (EOM) enlargement on computed tomography (CT) was not an exclusion criterion. An additional requirement was the ability to correctly complete the quality-of-life questionnaire and attend scheduled follow-up visits.

All procedures were performed under general anesthesia by the same surgical team, using an exclusive endoscopic transnasal approach. A complete ethmoidectomy was performed, with sphenoidotomy and removal of the lamina papyracea to expose the medial orbital wall. Two parallel anteroposterior incisions were made in the orbital periosteum, allowing controlled herniation of orbital contents into the paranasal sinuses. Periorbital fenestrations were conservative and standardized to minimize extraocular muscle imbalance. When indicated, decompression was extended to the roof of the maxillary sinus, while preserving the integrity of the infraorbital canal in all cases. No intraorbital fat resection was performed.

Proptosis was measured with the Hertel exophthalmometer (OCULUS Optikgeräte GmbH, Wetzlar, Germany), a clinical reference tool for quantifying exophthalmos, which has shown good correlation with radiological techniques such as CT [7,8]. Quality of life was assessed using the disease-specific GO-QoL questionnaire, validated in Spanish, which evaluates both the functional and appearance impact of the disease from the patient’s perspective [9,10]. GO-QoL was analyzed at the patient level. Hertel analyses were limited to within-orbit paired changes; no eye-level correlation modeling was performed.

The minimum clinical follow-up was 12 months. Postoperative assessments were scheduled at 2 weeks, 1 month, 3 months, 6 months, and 12 months, with annual follow-up thereafter. At each visit, complications, clinical evolution, proptosis measurements, and GO-QoL scores were recorded; the latter were obtained before surgery and at 12 months postoperatively.

For the analysis, means and standard deviations (SD) were calculated for preoperative and 12-month postoperative values of proptosis and quality of life. No a priori sample size calculation was performed; this was an exploratory consecutive series. We additionally report the mean paired difference with 95% confidence intervals (CIs) and the effect size (Cohen’s d for paired data, computed on the within-subject difference). Comparisons were performed using paired *t* tests (two-sided), with *p* < 0.05 considered significant. Statistical analyses were performed with Jamovi, version 2.4 (The Jamovi project, Sydney, Australia).

## 3. Results

### 3.1. Patient Characteristics

A total of 20 consecutive patients with inactive GO underwent pure endoscopic transnasal orbital decompression between 2020 and 2023. The cohort included 13 women (65%) and 7 men (35%), with a mean age of 49.4 years (range: 29–73).

### 3.2. Surgical Procedures and Follow-Up

In total, 22 procedures were performed involving 26 orbits: 14 unilateral cases; 2 bilateral cases operated in two separate sessions; and 4 bilateral cases operated in a single session. The minimum follow-up period was 12 months for all patients. A summary of pre- and 12-month outcomes is provided in Table 1.

### 3.3. Objective Outcomes (Proptosis)

**Figure 1:**
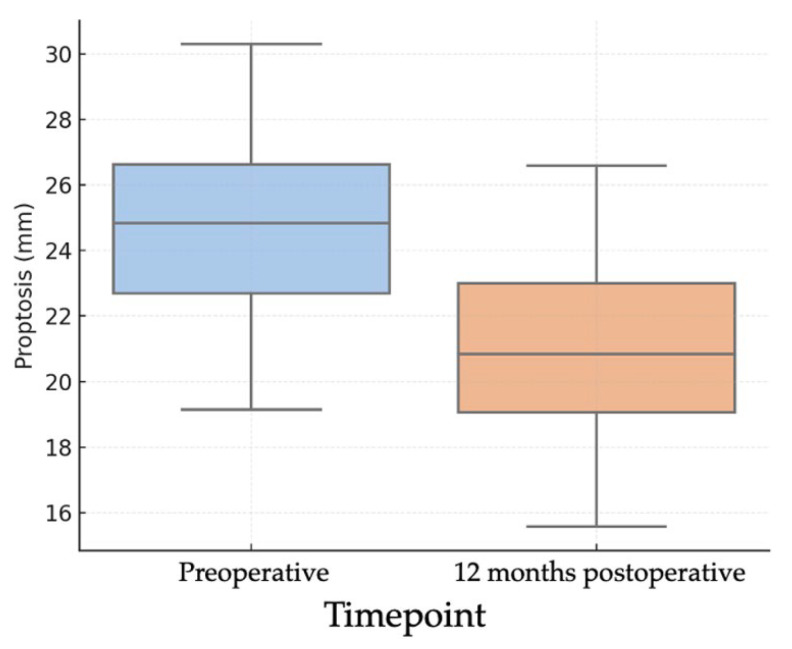
Boxplots of pre- and 12-month postoperative Hertel exophthalmometry (*n* = 26 orbits) showing median and interquartile range; whiskers denote 1.5 × IQR. Preoperative mean 24.2 ± 2.56 mm; postoperative mean 20.4 ± 2.10 mm; mean difference 3.85 mm (95% CI 3.03–4.68). Paired *t* test (two-sided), *p* < 0.001. Effect size: Cohen’s d (paired) ≈ 1.89 s.

**Figure 2:**
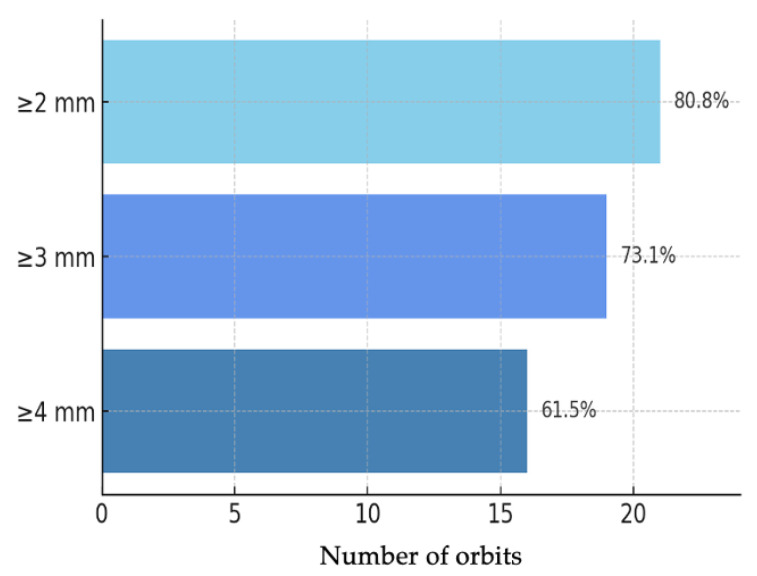
Bar chart of Hertel proptosis reduction at 12 months (*n* = 26 orbits): ≥2 mm: 21/26 (80.8%); ≥3 mm: 19/26 (73.1%); ≥4 mm: 16/26 (61.5%).

Objective evaluation of proptosis was performed using Hertel exophthalmometry in all 26 orbits. The mean preoperative proptosis was 24.2 ± 2.56 mm, while the postoperative mean was 20.4 ± 2.10 mm, corresponding to a mean reduction of 3.85 mm (95% CI: 3.03–4.68; *p* < 0.001) (Figure 1). This corresponded to a large effect size (Cohen’s d ≈ 1.89). This mean reduction represents the averaged effect of our standardized endoscopic protocol; the study was not powered for subgroup analyses by baseline proptosis or decompression extent. In the individual analysis of outcomes (Figure 2), 21 orbits (80.8%) achieved a reduction of at least 2 mm, 19 orbits (73.1%) achieved a reduction of at least 3 mm, and 16 orbits (61.5%) achieved a reduction of 4 mm or more.

### 3.4. Subjective Outcomes (Quality of Life)

**Figure 3:**
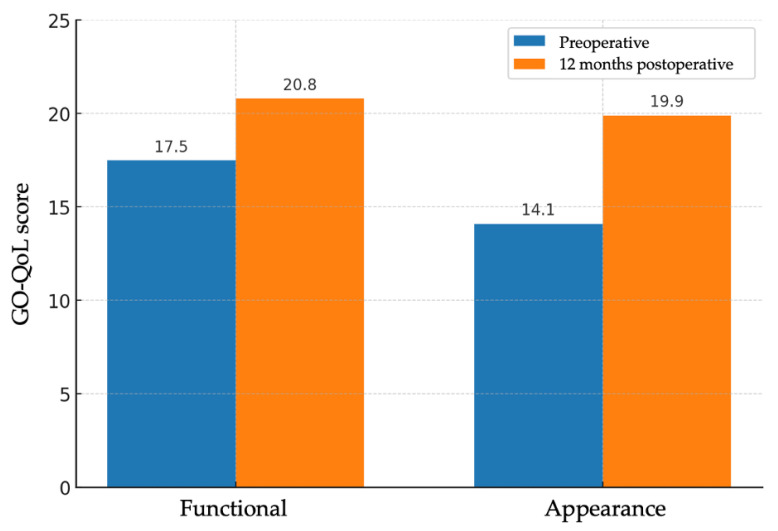
Bar charts of GO-QoL functional and appearance subscales before and 12 months after surgery (*n* = 22 patients); bars show means. Paired *t* test (two-sided): functional *p* < 0.001; appearance *p* < 0.001. Effect sizes (Cohen’s d, paired): functional ≈ 0.90; appearance ≈ 1.39.

Subjective evaluation of quality of life was performed using the GO-QoL questionnaire, which includes a functional subscale and an appearance subscale. A total of 22 valid questionnaire pairs were obtained (one per patient, except in sequential bilateral cases, in which a second form was administered after the contralateral procedure). On the functional scale, the mean score increased from 17.5 ± 3.95 points to 20.8 ± 3.81 points, with a mean improvement of 3.27 points (95% CI: 1.66–4.88; *p* < 0.001). On the appearance scale, the mean score increased from 14.1 ± 4.45 points to 19.9 ± 4.01 points, with a mean improvement of 5.77 points (95% CI: 3.94–7.61; *p* < 0.001) (Figure 3). The functional subscale showed a medium-to-large effect (Cohen’s d ≈ 0.90), and the appearance subscale a large effect (Cohen’s d ≈ 1.39). Despite statistical significance, mean GO-QoL changes did not reach established thresholds for clinically important improvement.

### 3.5. Complications

No intraoperative or postoperative complications were recorded, including new or worsened diplopia, nasolacrimal duct injury, optic neuropathy, or orbital hematoma. All procedures were performed as outpatient surgeries or with short hospital stays, and no patient required reoperation during the follow-up period.

## 4. Discussion

Our findings suggest that pure endoscopic transnasal orbital decompression in patients with inactive GO achieves a significant objective reduction in proptosis, as measured by Hertel exophthalmometry, with a mean decrease of 3.85 mm. This result is consistent with international series using similar techniques, such as those of Baeg (2023) and Guo (2024) [13,14], which reported mean reductions ranging from 3 to 5 mm with medial and inferomedial endoscopic approaches.

Pure endoscopic transnasal decompression provides direct access to the medial and inferomedial walls, avoids cutaneous incisions, and allows controlled management of sinonasal anatomy [15,16]. Balanced and lateral-wall techniques can yield greater exophthalmos reduction in selected patients but with different risk profiles, including new-onset diplopia and sensory changes [14,17,18]. Transconjunctival approaches offer scarless external cosmesis and reliable exposure of the medial wall; technique-specific factors such as periorbital opening and preservation of the inferomedial strut influence diplopia rates [19,20]. Procedure selection should be individualized according to baseline anatomy, disease stage, and patient priorities rather than a one-technique-fits-all model [14,15].

In addition to objective outcomes, we observed a statistically significant improvement in quality of life after 12 months, assessed with the GO-QoL questionnaire. However, the mean improvements (+3.3 points in the functional subscale and +5.8 in the appearance subscale) did not reach the minimal clinically important difference (MCID), estimated between 6 and 10 points for this instrument [9,10]. Modest improvements may still be meaningful for individual patients—particularly those with pronounced baseline disfigurement or higher concern about appearance—and should be acknowledged during preoperative counseling. Overall, these findings indicate a modest average patient-perceived benefit in inactive GO despite substantial exophthalmos reduction and should guide counseling to set realistic expectations, acknowledging that perceived benefit may lag behind anatomical change depending on baseline expectations and chronicity.

The subjective assessment of GO has gained increasing relevance in recent years, with recommendations that therapeutic decisions should integrate both objective measures and patient-reported perception [21]. The GO-QoL, validated and endorsed by major guidelines [2], captures dimensions not reflected in anatomical measurements, such as social discomfort, self-image, or visual fatigue.

Reports have described an association between exophthalmos reduction and improved esthetic perception, although the relationship is not necessarily proportional across individuals [11,12]. This indicates that, although some relationship exists, an objective reduction in exophthalmos does not necessarily translate into a proportional improvement in patient-perceived quality of life. This underscores the complexity of the disease and the need for individualized approaches.

From a technical perspective, our series employed a pure endoscopic technique, without external approaches or intraorbital fat removal. This strategy has been associated with lower complication rates compared with combined or fat-removal techniques [19], particularly regarding new-onset diplopia or orbital hematoma. In our study, no major complications were observed, supporting the notion that this approach can be considered safe when performed by experienced teams. Furthermore, recent reviews have highlighted the safety and efficacy of endoscopic decompression of the medial and inferomedial walls in selected cases [18]. Similarly, the retrospective series of Woods (2020) also reported consistent outcomes regarding the effectiveness and safety of this minimally invasive approach [16].

This study is retrospective and single-center, with a modest sample size and no control cohort, which limits generalizability. No a priori power calculation was undertaken; findings should be interpreted as hypothesis-generating. EOM enlargement was not quantified volumetrically, and the study was neither powered nor preplanned for subgroup analyses by baseline proptosis or by medial versus inferomedial extent. Within-patient (bilateral) eye correlation was not modeled, which may underestimate variance and increase the risk of a Type I error for eye-level outcomes; results are therefore presented at the aggregated level. These factors may have contributed to the lack of clinically meaningful changes in GO-QoL. Possible explanations for sub-MCID mean changes include near-ceiling baseline GO-QoL scores in some patients, heterogeneity in expectations and chronicity, and the modest sample size, which may attenuate average perceived gains in the inactive phase. We did not compute correlations between anatomical and patient-reported changes because individual-level paired data suitable for that analysis were not available within the prespecified dataset; this should be explored in future studies. Nevertheless, the minimum 12-month follow-up, the homogeneous pure endoscopic technique, and the systematic data collection strengthen the reliability of our findings.

To the best of our knowledge, this study is among the few to combine objective and subjective evaluation of GO after pure endoscopic orbital decompression. While the data come from a single national center, they provide valuable evidence that complements the limited international literature. Future research should validate these findings in prospective, multicenter cohorts with predefined stratification by baseline severity and include objective volumetric CT or magnetic resonance imaging metrics. The incorporation of psychometric instruments beyond GO-QoL, together with comparative analyses across techniques, may better capture subtler patient-perceived benefits and refine selection and counseling.

## 5. Conclusions

Our results support the use of pure endoscopic transnasal orbital decompression as a safe and effective option for patients with inactive GO, being associated with a significant objective reduction in proptosis and perceived functional and esthetic improvement. However, the relationship between anatomical changes and patient-reported perception is not always linear, underscoring the importance of integrating both clinical parameters and quality-of-life measures when evaluating surgical outcomes. Larger studies with longer follow-up are needed to optimize candidate selection and to personalize treatment according to individual expectations and needs.

## Figures and Tables

**Table 1:** Summary of primary outcomes (preoperative vs. 12 months). Values are mean ± SD. Mean difference is the paired pre–post change with 95% CI. *p* from two-sided paired *t* tests. Effect size is Cohen’s d for paired data.

Outcome	Sample	Preoperative (Mean ± SD)	12 Months (Mean ± SD)	Mean Difference	95% CI	*p* Value	Cohen’s d (Paired)
Hertel exophthalmometry (mm)	*n* = 26 orbits	24.2 ± 2.56	20.4 ± 2.10	3.85	3.03–4.68	*p* < 0.001	1.89
GO-QoL functional (points)	*n* = 22 patients	17.5 ± 3.95	20.8 ± 3.81	3.27	1.66–4.88	*p* < 0.001	0.90
GO-QoL appearance (points)	*n* = 22 patients	14.1 ± 4.45	19.9 ± 4.01	5.77	3.94–7.61	*p* < 0.001	1.39

## Data Availability

The original contributions presented in this study are included in the article. Further inquiries can be directed to the corresponding author.

## Abbreviations

The following abbreviations are used in this manuscript:

GO	Graves’ orbitopathy
GO-QoL	Graves’ Ophthalmopathy Quality of Life
EOM	Extraocular muscle
CT	Computed tomography
SD	Standard deviations
CIs	Confidence intervals
MCID	Minimal clinically important difference
